# Periodic Fractal-Growth Branching to Nano-Structured Grating Aggregation in Phthalic Acid

**DOI:** 10.1038/s41598-020-60782-0

**Published:** 2020-03-04

**Authors:** Tzu-Yu Chen, Eamor M. Woo, Selvaraj Nagarajan

**Affiliations:** Department of Chemical Engineering, National Cheng Kung University, No. 1, University Road, Tainan, 701-01 Taiwan

**Keywords:** Nanoscale materials, Soft materials, Materials science

## Abstract

Small-molecule phthalic acid (PA), confined in micrometer thin films, was crystallized in the presence of strongly interacting tannic acid (TA) to investigate crystal assembly and correlation between banded patterns and branching structures. Several compositions of the mixture of ethanol/water solutions and evaporation temperatures were also manipulated to investigate the kinetic effects on the morphology of PA crystals. With increasing evaporation rate, the morphology of PA crystals systematically changes from circular-banded spherulites to highly ordered grating-banded patterns. A unique periodic fractal-branch pattern with contrasted birefringent bands exists at intermediate evaporation rate, and this unique grating architecture has never been found in other banded crystals. Crystal assembly of these three periodic morphologies was analyzed by utilizing atomic-force microscopy (AFM) and scanning electron microscopy (SEM) to reveal the mechanisms of formation of hierarchical structures of PA. The detailed growth mechanisms of the novel fractal-branching assembly into circular- or grating-banded patterns are analyzed in this work.

## Introduction

Cooling of a matter in liquid state causes it to crystallize into either 2D or 3D ordered states. The crystallization process may occur in sequential steps to gradually increase the degree of order while in the meantime the hierarchical aggregation structures may diversify in patterns and vary significantly in increasingly higher-order morphologies. Crystallization of atoms or molecular compounds into ordered states has been a complex yet fascinating phenomenon that has attracted extensive scientific studies from classical to modern times, to cite just a few recent examples^1–3^. Self-assembly of organic or inorganic compounds, of either small-molecules or polymers, has been interesting and intriguing, yet complex issues, in soft matters either synthetic or natural. Fractal patterns are widely seen in hierarchical structural assemblies in natural materials. Khire and Yadavalli, *et al*.^4^ by using high-resolution atomic-force microscopy (AFM), investigated the fractal self-assembly of silk protein sericin [a protein by *Bombyx mori* (silkworms) in producing silk], and observed the fractal structure of the silk protein’s self-assembly, where they proposed that the protein globules may be thought to act as seeds for the subsequent attachment of protein molecules and the formation of the fractal architectures. Guo *et al*.^5^ studied the relationship between the fractal-ladder viscoelastic behavior of bio-fibers with fractal ladder hyper-cell structures. Snowflakes or aggregates of self-similar snow/ice single crystals are well known to be an excellent example of fractal patterns in nature. Yang, *et al*.^6^ proposed the fractal growth kinematics of snow-flakes with many self-similar multilevel small structures. Crystalline morphology is governed by the cooperation of the driving force of crystallization and the transport of atom, ions, molecules, and heat.^7^ When the growth condition is relatively far from equilibrium, meaning the increase of driving force, random polycrystals may aggregate into an entity displaying spherulitic morphology. On the other hand, when the growth condition is relatively near equilibrium, meaning the decrease of driving force, dendritic morphology with crystallographic branching appears. Dendritic growth with hierarchical structure has attracted much attention for the effect on the corresponding physicochemical properties. Introducing the gelatin matrix is a common method to control the nucleation and induce dendritic morphology for crystal developing from the evaporation of solvent. Many inorganic compounds and metal salts have been reported to form highly symmetric branching structures under appropriate conditions^8–10^. Besides, semi-crystalline polymers diluted with another strongly-interacting amorphous polymer were also found to have the ability to form well-defined dendritic patterns^11–15^. Tannic acid (TA), with multi-phenol groups, has been reported to have a strong interaction with some semi-crystalline polymers with carbonyl groups and further induce diversified morphologies^11–13^. With the diluent effect of TA, the morphology of poly(ethylene oxide) (PEO) transformed from typical spherulites with normal Maltese-cross extinction into feather-like dendritic spherulites. It is clear that the addition of TA effectively modulates the crystallizing tendency of PEO and thus induce different types of morphologies owing to the stronger interactions between PEO and TA^11^. Besides, poly(ethylene succinate) (PESu) also undergo a similar transformation. With increasing the composition of TA, the morphology of PESu transformed from well-defined round spherulites into seaweed-like dendritic spherulites composed of lenticular-shaped single crystals, indicating that the strong H-bonding interaction between TA and PESu was capable of inducing dendritic morphology consisting of ordered and regular single crystal assembly^12,13^.

Ring-banded pattern with periodic assembly in the crystallization of polycrystals is another hierarchical structure, which also has been investigated extensively. Periodic ring patterns are commonly observed in some long-chain polymers^16–24^ and many small-molecule compounds^25–30^. Both dendritic pattern (grating structure) and ring-banded pattern are highly ordered self-organization in crystallization. However, the correlation between these two kinds of hierarchical structures does not have an adequate understanding yet. Woo *et al*.^22^ investigated the interior lamellar assembly of poly(dodecamethylene terephthalate) (PDoT or P12T) banded spherulites in bulk sample and discovered that the P12T banded spherulites revealed corrugated-board (onion-like) structures, composed of tangential and radial lamellae intersecting at about 90° angle. Moreover, tangential crystals generally fanning out from the bottom and further evolving branches in radial directions were observed with careful inspection. The periodicity of branching is about 5 μm, which completely matches with the optical band spacing. The branching of tangential and radial lamellae and periodic perpendicular intersection result in multi-shell assemblies thereby lead to the optical patterns of periodic rings. Thus, the fact clearly suggests that there must be some correlation between the grating assembly and periodic banded pattern, which needs more systematic explorations to get a comprehensive elucidation for highly organized structures.

The kinetics of solvent evaporation is one of the important factors influencing the crystal packing and the final morphology when a compound crystallizes from solutions. For example, poly(l-lactic acid) (PLLA) can reveal a wide variety of morphologies with complex optical birefringence patterns depending on the evaporation temperature and the evaporation rate, as reported in recent research^24^. Phthalic acid (PA), a small-molecule organic compound, has been reported having the ability to form diversified morphologies via rapid evaporation-induced crystallization from PA solutions (usually in water/ethanol mixtures), which is governed by the evaporation conditions including the type of solvent, composition of the solvent mixture, and evaporation temperature^29^. At low humidity (15%), PA can form non-crystallographic branched dendrites at the edge of specimens^31^.

In the present work, PA, blended with a strongly-interacting diluent TA (via inter-molecular –OH hydrogen bonding), was crystallized under controlled evaporation conditions to induce diversified morphology, including ring-banded, periodic branching and grating patterns. Tannic acid (TA) has multiple –OH groups per molecule; thus, it has strong capacity to interact with PA that has two carboxylic groups on meta-positions of the benzene ring. The effect of diluents, evaporation temperature, and the compositions of mixtures of solvents are systematically examined to clarify the modulation factors governing crystal assembly into final morphologies with periodic patterns. Careful inspection of the assembly in the diversified PA crystal aggregations aimed to reveal mechanisms of periodicity in crystal habits. Eventually, the correlation between the banded patterns and grating assembly via fractal growth can be established.

## Results and Discussion

### Morphology evolution of PA/TA system

**Figure 1:**
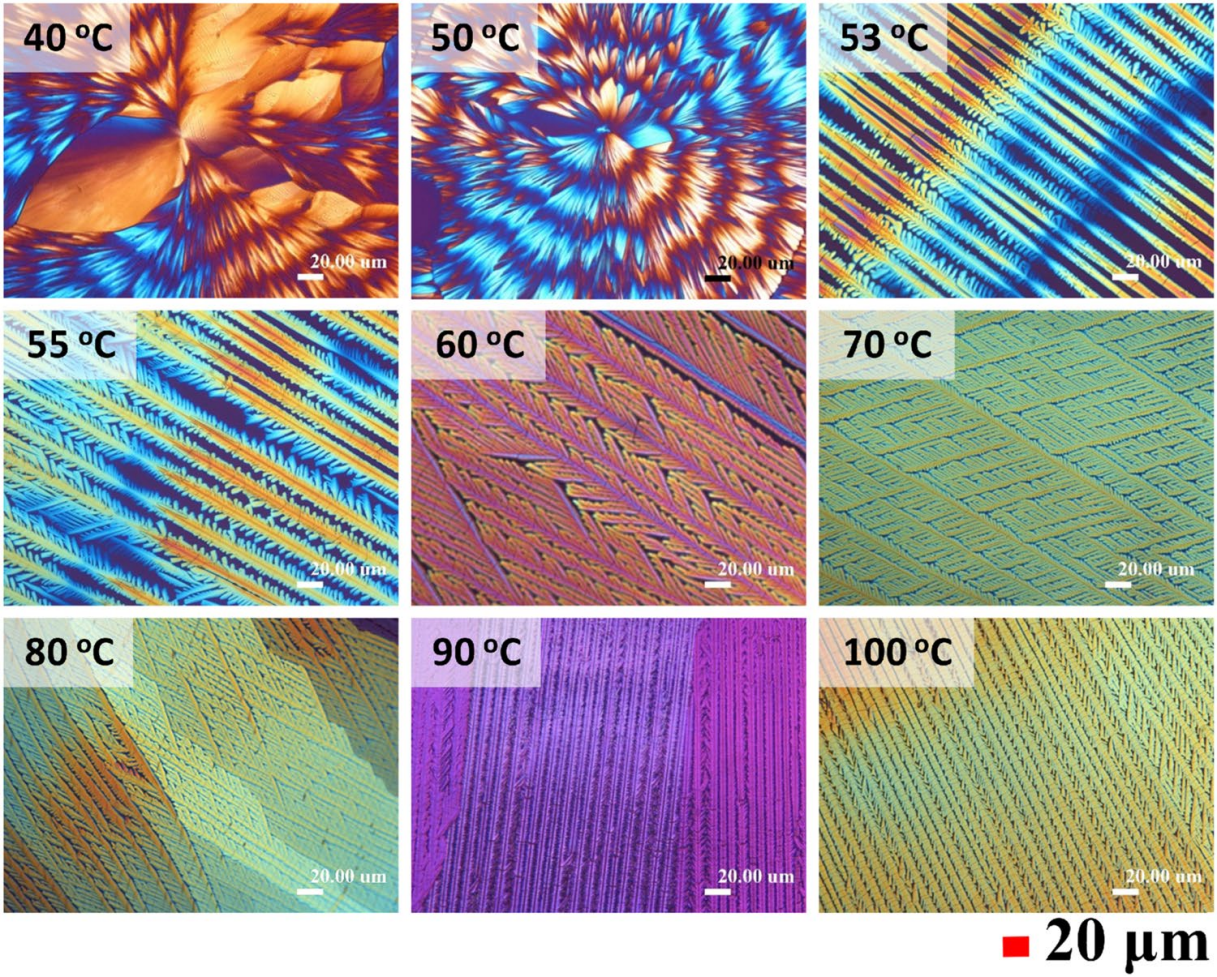
POM graphs of PA/TA (80/20 wt. ratio) dissolved in ethanol/water (20/80 vol. ratio) co-solvents and then evaporated to crystallize at various *T*_*c*_ as indicated on graphs [scale bar = 20 μm].

Evaporation conditions, including evaporation temperature and the compositions of mixtures of solvents (water-ethanol), have been reported to play an important role in the crystallization of PA, which might influence the crystallization kinetics and thus final morphology of PA crystals^29^. Accordingly, the evaporation conditions of PA/TA (80/20) mixture were manipulated in several possible combinations to induce a wide varieties of morphologies for respective analysis. Figure 1 shows POM micrographs of PA/TA (80/20) blend dissolved in ethanol/water (20/80) solution and then evaporated to crystallize at *T*_*c*_ = 40–100 °C, displaying diversified morphologies of PA crystals with respect to *T*_*c*_’s. At low *T*_*c*_ (40–50 °C), PA crystals reveal ring-banded spherulites with periodic variation of optical birefringence (dark/bright).

Nevertheless, at higher *T*_*c*_ (60–100 °C), the morphology changes into grating structures with well-organized branches, and every branch mutually intersects with main stalks at angles of approximately 60°. Moreover, with increasing evaporation temperature, the grating structures become more compact and the dendrites become finer and thinner. Surprisingly, at the intermediate *T*_*c*_’s (53–55 °C), a novel periodic-branch banded structure with contrasted birefringent patterns was found. Periodic-branch banded structures are composed of main stalks which display the positive optical birefringence (orange colour), and periodically spawned branching dendrites which display the negative birefringence (blue colour). All main stalks are parallel and grow continually forward along the same direction, and the branching dendrite aggregations periodically emerge at a specific interval, leading to a grating structure that display an optical banded pattern. It is obvious that the PA crystals display three categories of diversified grating morphologies depending on the evaporation temperatures (40–100 °C). These three categories are: (I) circularly ring banded at *T*_*c*_ = 50 °C, (II) grating structures with fractal branches whose periodic crossbar pitches increasing with *T*_*c*_ (*T*_*c*_ = 53–55 °C), and (III) fully grating structures with infinite crossbar pitches (*T*_*c*_ = 60–100 °C).

In addition to PA/TA = 80/20, several other compositions of PA/TA mixtures crystallized at *T*_*c*_ = 70 °C were also examined as shown in ESI Fig. S1. The POM micrographs for PA/TA mixtures of various compositions of 100/0, 90/10, 80/20, and 70/30, all crystallized at same *T*_*c*_ = 70 °C, prove that increase of TA content in the PA/TA mixtures has an equivalent effect of increasing *T*_*c*_ to lead to grating architectures in PA crystals. Neat PA (with no TA) shows ordinary alternating bright/dark circular rings patterns as widely reported earlier in the literature^29,30^. However, the crystalline morphology of PA/TA mixtures changes to a grating pattern upon crystallizing with TA, which is novel and has not been reported before. The patterns appear are highly influenced by composition of PA/TA mixtures; however, a few specific compositions (e.g., PA/TA = 80/20) appear to lead to highly ordered grating structures. To analyse the mechanisms of formation of such unique crystalline morphologies and the lamellar crystal packing and aggregation into periodically ring-banded and grating structures, the PA/TA = 80/20 (wt. ratio) composition was chosen as a main focus of examination, with morphologies of other compositions for comparison.

**Figure 2:**
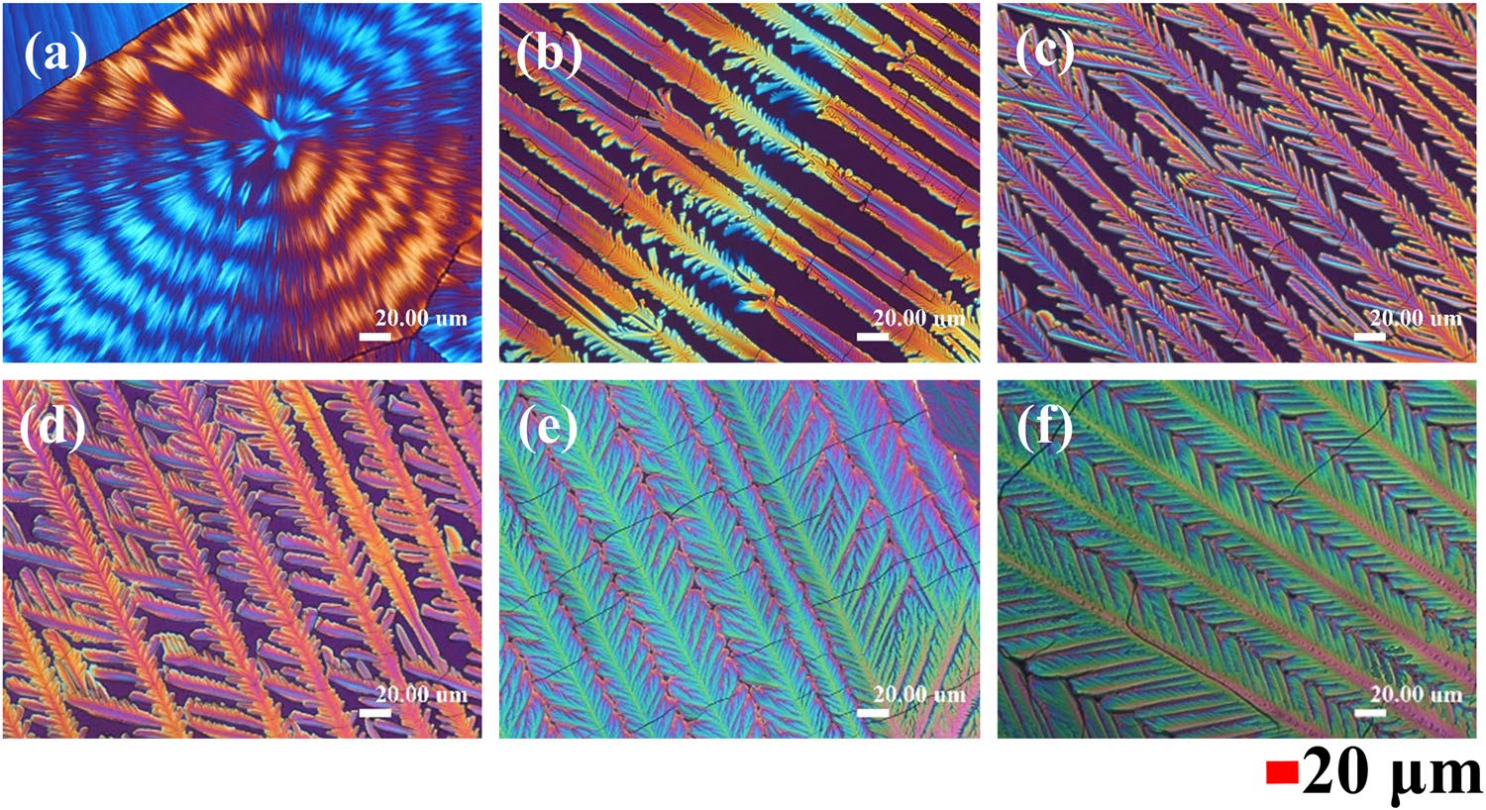
POM graphs of PA/TA (80/20 wt. ratio) blend dissolved in different compositions of ethanol/water solution (vol. ratio): (**a**) 40/60, (**b**) 45/55, (**c**) 50/50, (**d**) 60/40, (**e**) 70/30, and (**f**) 80/20, and then evaporated to crystallize at fixed *T*_*c*_ = 40 °C. [Scale bar = 20 μm].

In order to clarify the effect of solvents on the PA crystalline morphology, various compositions of the mixed ethanol/water solution were used as co-solvents of PA to develop the final crystallized specimens. Figure 2 shows POM graphs of PA/TA (80/20) mixture dissolved in different compositions of the ethanol/water solution, and then evaporated to crystallize at a fixed *T*_*c*_ = 40 °C. When the content of ethanol in the mixed solvent is relatively low (40 vol.%), only ring-banded PA spherulites can be observed. However, once the composition of ethanol of the solvent is above 50 vol.%, grating patterns are formed upon solvent evaporation. By increasing the content of ethanol in the mixed solvent, the volatility of the co-solvent at a fixed *T*_*c*_ is enhanced, which leads to that the grating structures gradually become more compact with finer dendrites. Note that the periodic-branch banded structures can form only in the intermediate compositions of the co-solvent with ethanol being ~45 vol.%. The two extremes of PA morphologies are the circularly ring-banded (at lower evaporation rates of co-solvent) and fully grating structures (very fast evaporation rates).

The POM graphs in Fig. 2 show variety of fractal-grating patterns, except for Fig. 2a, which is circularly ring-banded. The fractal dimension (D) of fractals, a unit-less measure of the extent of fractals as defined earlier by Lin, *et al*.^32^ and similarly by other investigators^33^, could be estimated from the binary (i.e., B&W) POM images of the fractal-grating structures of PA/TA system (ESI Fig. S2); and the results of D for each fractal patterns could be obtained from the slopes of plots of ln(N(λ)/ln(λ), which is shown in ESI Fig. S3. The calculated values of the fractal dimension (D)^32,33^ of the fractal-grating structures for PA crystallized with different composition of the ethanol/water solution of varying volume ratios of 45/55, 50/50, 60/40, 70/30 and 80/20, respectively, were estimated as 1.824, 1.881, 1.901, 1.917 and 1.868, respectively. Within statistical confidence level, the fractal dimension of PA gratings increases slightly with the ethanol volume ratio in the co-solvent. A bigger jump of D quantity exists in PA cast from ethanol/water = 45/55 vs. 50/50, which is understandable as the fractal patterns in Fig. 2b,c (cast from ethanol/water = 45/55, 50/50, respectively) differ more significantly; however, fractals of Fig. 2c to Fig. 2f (cast from ethanol/water = 50/50, 60/40, 70/30 and 80/20, respectively) share more similarity in their grating fractal patterns.

**Figure 3:**
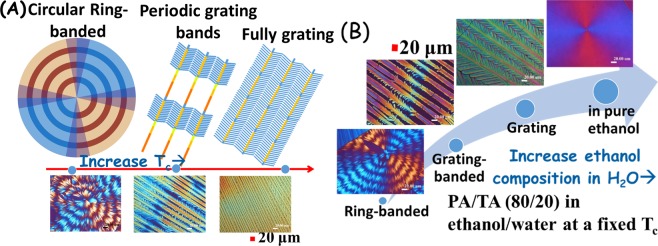
Morphological evolution of solvent-evaporated PA/TA (80/20) crystals from circular-ring banded to grating-like increasing evaporation rates: (**A**) by increasing casting T_c_, (**B**) by increasing evaporation rate with adjusting the ratio of water/ethanol solvents.

With respect to increasing the evaporation temperature or increasing the TA composition, or the ethanol ratios in the mixture of ethanol/water, the morphology of PA crystals undergoes a steady evolution from one pattern to another. The fact suggests that the crystalline morphology of PA/TA (80/20) mixture is sensitively modulated and governed by the solvent evaporation rate. Note the solvent evaporation rate is associated with the ratios of ethanol/water, where a higher ratio of ethanol in the mixture registers a higher evaporation rate at a specific temperature. The morphological evolution of PA/TA (80/20) blend with increasing the evaporation rate is schematically summarized in Fig. 3. Under low solvent-evaporation rates, the morphology displays ring-banded spherulites. By comparison, under an intermediate evaporation rate, PA crystals show a unique periodic-branched banded pattern. Under a high evaporation rate, the morphology changes into grating patterns. Trend of variation in PA morphology is obvious with respect to the evaporation rate.

Lower evaporation leads to circular ring-banded pattern, conversely, higher rate leads to loosely-packed grating structures of various crossbar pitches. During solvent evaporation, the rate of change in the degrees of super-saturation (PA conc. in solvents) apparently is associated with both temperature and co-solvent ratio (water/ethanol). Higher temperature of casting or higher ratio of ethanol in the water/ethanol co-solvent leads to a higher rate of evaporation; thus, either of these two factors similarly results in a higher changing rate of super-saturation. Such higher changing rate in super-saturation universally leads to altering the originally circularly banded lamella assembly to grating-banded, then to fully grating architectures.

### Ring-banded spherulites crystallized at lower temperatures

**Figure 4:**
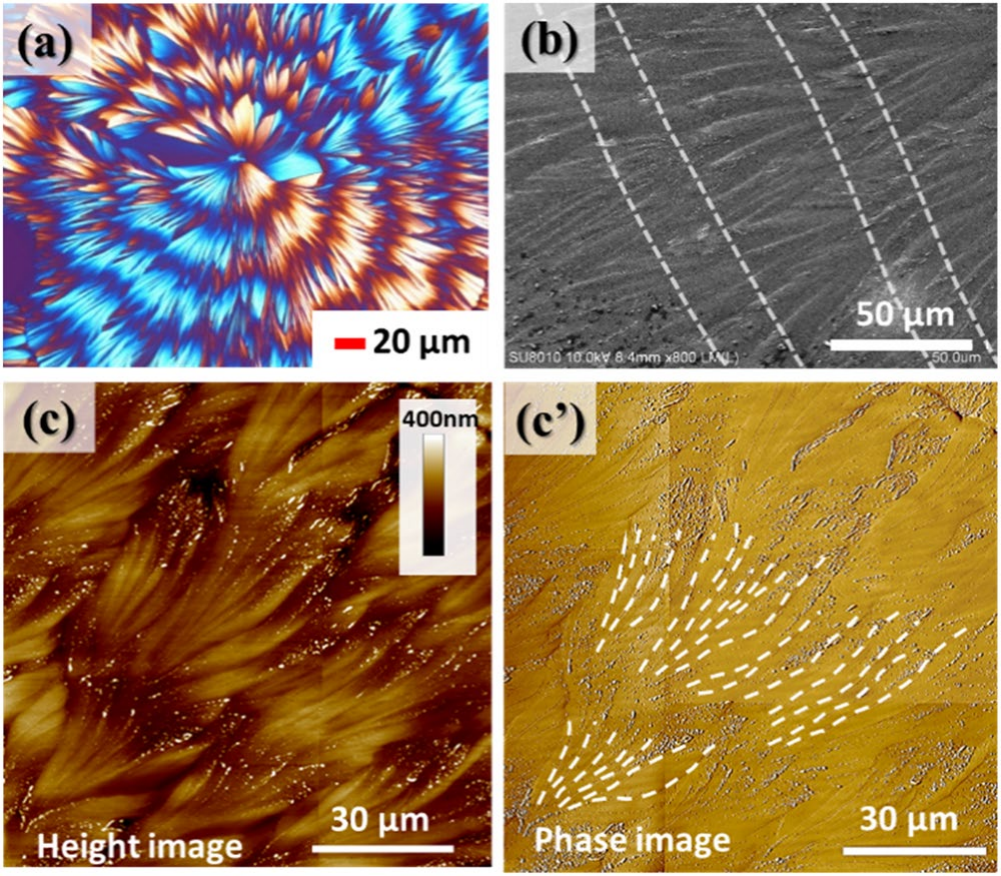
(**a**) POM graph of PA/TA (80/20) dissolved in ethanol/water (20/80) crystallized at 50 °C, (**b**) SEM micrograph, (**c**) AFM height image and (c’) AFM phase image for the top surface of circular ring-banded pattern of PA/TA (80/20) crystallized at 50 °C.

As shown earlier, the PA crystalline morphology goes through a systematic transition with respect to increasing evaporation temperature. Thus, PA/TA (80/20) blends crystallized at Tc = 50, 53, and 60 °C are chosen to in-depth analysis the crystal assembly of ring-banded, periodic fractal-branch banded, and grating architectures, respectively. Figure 4 shows (a) POM micrograph, (b) SEM micrograph, (c) AFM height image and (c’) AFM phase image for the top surface of ring-banded pattern of PA/TA (80/20) crystallized at 50 °C. The POM image reveals alternate dark/bright banded patterns with some zigzag irregularity owing to the branching structure, where branches grow to different length dimensions. The SEM graph provides clear evidence for numerous crystal branches periodically developing and dividing along the radial direction of the spherulites. Moreover, AFM images reveal that the distinct large branches region is the ridge while the region which is seemingly flat without texture of lamellae constitutes the valley. The band spacing is about 40 μm, as revealed in the POM graph of Fig. 4a and in agreement with the AFM images of Fig. 4c. With careful inspection, one can observe that as the crystals evolve, branches gradually fan out and eventually turn into the sharp needle-like end and are submerged and hidden underneath the end of ridges. In addition, some branches bend away from the radial direction due to the impingement with neighbouring branches, leading to some irregularity of branching patterns thus forming the optical zigzag bands.

**Figure 5:**
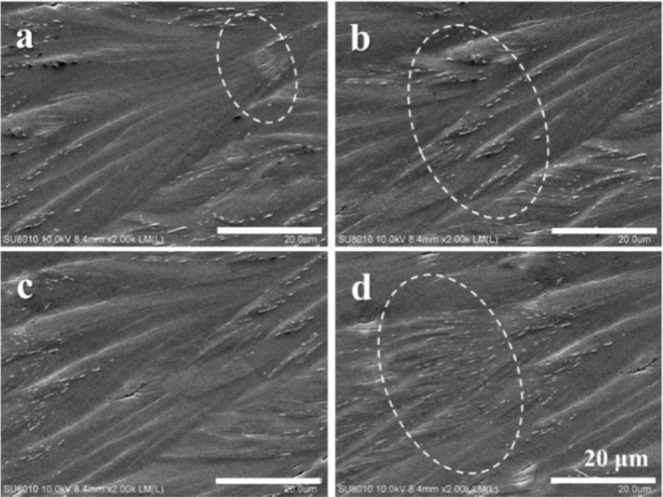
(**a**–**d**) SEM micrographs of PA/TA (80/20) blend dissolved in ethanol/water (20/80) crystallized at *T*_*c*_ = 50 °C. The circle regions are the valleys of the banded spherulites, showing compact structures with tiny branches that have been re-oriented to different directions.

Figure 5 shows SEM micrographs of the top surface of PA/TA (80/20) blend crystallized at 50 °C, revealing the detailed crystalline assembly of the ring-banded spherulites. The circled regions marked in the SEM micrographs show that the valley regions composed of compact structures with tiny branches. With the development of the spherulites, these tiny branches radiate out along the radial direction with slightly bending and meanwhile emerge upward and expand, resulting in the fan-like crystals in the ridge region. Then the fan-like crystals gradually taper into needle-like ends and submerge into the valley region, where these crystals in the valley then grow in next cycle in fractal pattern into more branches and thicken into main stalks in the next cycle. The process is repeated in same cycle, leading to the observed periodically fractal-branching crystalline structure. That is to say, the periodic banding in the PA spherulites (crystallized from PA-TA mixtures) is not due to lamellae helix-twist from the nucleus centre continuously to periphery, but rather due to periodic fractal growth of crystal branches in the ridge accompanied with crystal re-orientation in the valley region. The periodicity of branching is 40 μm, which completely matches with the band spacing observed from the AFM and POM results.

**Figure 6:**
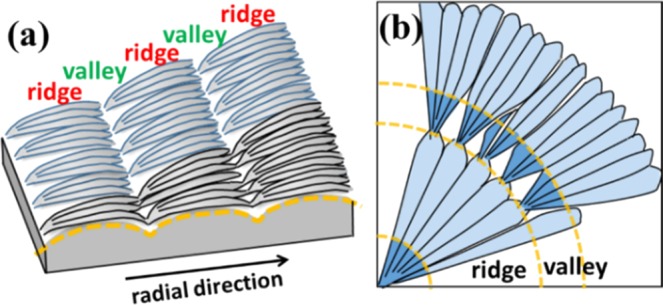
Schematic illustration representing (**a**) key characteristic of the top surface and (**b**) corresponding optical patterns of ring-banded pattern of PA/TA (80/20) dissolved in ethanol/water (20/80) co-solvent and crystallized at 50 °C.

The main characteristics of the top surface and corresponding optical patterns of the ring-banded PA spherulites in PA/TA (80/20) crystallized at 50 °C are schematically illustrated in two cartoons of Fig. 6(a,b) for the lateral view and top view, respectively, of the fractal growth. Figure 6a shows crystal bundles initially packed compactly evolve into 3–4 tiny branches, and subsequently emerge along the radial direction to form 3–4 obvious fan-like crystals in the ridge. Meanwhile, the fan-like crystals expand within the circumferential direction till final impinging with neighbouring crystals to fill the space in the ridge region. After crossing the ridge region, each of the fan-like crystals tapers into sharp needle-like ends and bend and sink in synchronizing pace into the valley. It is worthy to point out that every needle-like end will further form 3–4 tiny branches in the next cycle, which repeats again and again till draining of all molten species. The compact structures consisting of tiny branches lead to the observed flat area in the valley. The fan-like crystals with crevices at the interface of adjoining crystals lead to the obvious relief pattern in the ridge. The periodic branching assemblies display the surface-relief patterns on the surface. Note that the bending of fan-like crystals observed in the ridge with seemingly twist phenomena might have led researchers to misinterpret the formation mechanisms of banded spherulites; however, the fact is that orderly branch growth is the key characteristic of many banded spherulites. As addressed, periodic growth with branching is needed to fill the ever expanding space in an expanding spherulite. Figure 6(b) shows the correlation between the branching assembly and optical patterns. In the ridge region, fan-like crystals assemble linearly along the radial direction, resulting in the bright bands. At the end of the fan-like crystals, the crystals shrink into sharp needle-like ends and tilt away from the original direction, and further develop tiny branches with compact structures, resulting in the dark bands in the valley. The periodic branching, accompanied by intermittent branch spraying/tilting away from the radial direction, is the main crystal assembly mechanisms responsible for the alternating birefringence (bright/dark) contrasts.

### Fractal-branch banded patterns crystallized at intermediate temperature

**Figure 7:**
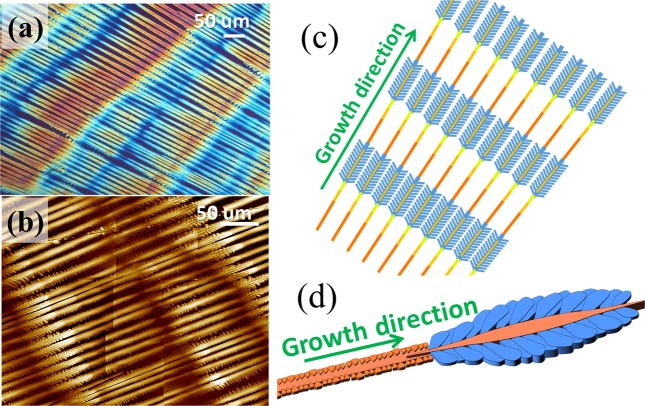
(**a**) POM, (**b**) AFM micrographs, and (**c**) schematic illustration representing the main characteristic corresponding to birefringence of the periodic fractal-branch banded pattern, (**d**) single main stalk of PA/TA (80/20) dissolved in ethanol/water (20/80) crystallized at 53 °C. [POM scale bar = 50 μm].

Crystallization of PA by solvent evaporation at temperatures higher than 50 °C was also analysed. It has been shown in the previous discussion that PA/TA (80/20) blend may display diversified morphologies as modulated by the solvent evaporation rate. Under a specific condition, PA/TA (80/20) blend can be crystallized into unique periodic fractal-branch banded patterns, which have never been reported in ordinary banded spherulites. Therefore, it is worthy to explore this highly ordered hierarchical structure to reach a more comprehensive understanding of the formation mechanisms of periodic bands. Figure 7(a) shows POM image of the periodic fractal-branch banded pattern, revealing alternatively contrasted birefringence (orange/blue) bands with a period of ca. 250 μm (or “crossbar pitch” in a grating structure), which is much larger than the band spacing of banded spherulites. In addition, these unique fractal-branch banded structures develop in a parallel direction instead of radiating out from the centre to periphery. Apparently, the formation mechanism of these periodic fractal-branch banded patterns, composed of many repeatedly multiple fractals, is quite distinct from the regular circularly-banded spherulites. A “periodic fractional section” of the fractal can be divided into two regions: (1) straight main stalk and (2) fernlike dendrites. During the crystallization process, the main stalk grows almost linearly forward till a specific length of 150μm, then starts to branch out as fernlike dendrites from the main stalk for length of 100μm, where the fernlike dendrites are perpendicular to the main stalk (mutually intersected at 90° angle). The periodic development of fernlike dendrites leads to the crystal-dense bands, and the main stalk region without obvious branches is the seemly crystal-poor bands. In addition, the top surface observation of PA/TA (80/20) blend crystalized at *T*_*c*_ = 53 °C evidently indicates the periodic branching patterns, as shown in Fig. 7(b), whose main characteristic of the fractal-branch banded pattern is schematically illustrated as a crossbar grating structure in Fig. 7(c). The linear stalks without branches grow straight forwardly revealing optical positive birefringence (orange colour); by comparison, the fernlike dendrites grow perpendicularly to the main stalks, revealing optical negative birefringence (blue colour), with the main stalks remaining orange-colour birefringence. The contrast birefringence obviously is due to crystal orientation intersecting at ca. 90° angle to each other. Hence, the periodic branching of the fernlike dendrites accompanied with the formation of contrasted birefringence leads to the periodic fractal-branch banded pattern that is actually a grating structure whose crossbar pitch matches with the optical band spacing. Figure 7(d) shows zoom-in scheme of an individual lamellar stalk in the grating-like mesh structure that forms alternate optical grating bands as viewed in POM with a tint plate. The cycle repeats with a zone of branchless growth then hefty-branching zone packed by fern-leaf-like pattern, until draining out. These two zones together constitute a “crossbar pitch”, whose total length dimension is exactly equal to one optical banding of ca.100 + 250 = 250 μm.

**Figure 8:**
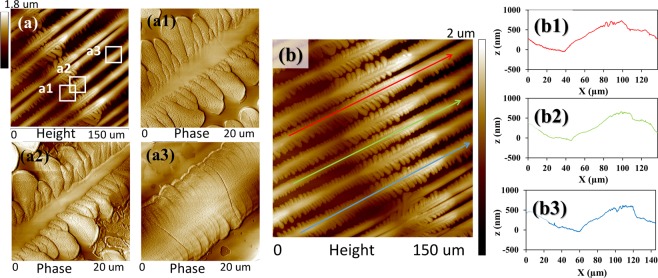
AFM micrographs (**a**,a1,a2,a3) height images, and (**b**,b1,b2,b3) zoom-in phase images of periodic fractal-branch banded pattern of PA/TA (80/20) dissolved in ethanol/water (20/80) co-solvent and crystallized at 53 °C.

In order to further investigate crystal assembly of the fractal-branch banded pattern, AFM characterization was conducted. Figure 8 shows AFM height image, zoom-in phase images, and height profiles of periodic grating-banded pattern of PA/TA (80/20) crystallized at 53 °C. The height image also reveals that the thickness of the crystalline structures in the branch-poor-stalk region is lower than those in the main stalks with fernlike-dendrite branch-rich region. Furthermore, POM and AFM analyses clearly indicate that the branch-poor stalk is the valley and the main stalk with fernlike dendrites is the ridge in the fractal-branch banded pattern, as shown in Fig. 8(a). This structure may result from the periodic change in concentration gradient. When the material is adequate, fernlike dendrites with plane crystals can grow from the main stalk in the ridge. By contrast, with lack of any crystallisable substances, the stalk in the valley exhibits a linear stalk without branches. In the fernlike dendrites, tiny branches initially grow loosely from the flat plateau-like main stalks and subsequently expand to fill the available space till impingement with the neighbouring crystals, resulting in the obvious sector-like planes, as shown in Figs. 8(a1,a2). On the other hand, on the lateral sides of the stalk, tiny branches grow intensively with a compact structure. Hence, there is no sufficient space for branches to grow in dimension, resulting in the small needle-like branches, as shown in Fig. 8(a3). Figure 8b1–b3 shows AFM height profiles along three different radial stalks, and all three analyses consistently reveal that the rimmed stalk constitutes the valley band (lower region), and the fern-like branching constitutes the ridge band (higher region). The height difference (valley to ridge) is ca. 600–800 nm.

Besides, more detailed AFM analysis focusing on different regions of fractal-branch banded pattern was also executed. For brevity, data are placed in ESI Fig. S4 to show AFM height images and height profiles of (a) fernlike dendritic region and (b) stalk region of periodic fractal-branch banded pattern. The fernlike dendrites regions are composed of large sector-like plane branching from flat plateau-like main stalks. The stalk regions show tiny needle-like branches growing compactly but small in dimension, thus revealing a macroscopically branch-poor structure. The zoom-in AFM phase images show clear crystal assembly of the fern-like dendrites and stalk regions. As discussed, the POM micrographs show optical birefringence periodic bands in PA/TA (80/20) crystallized at *T*_*c*_ = 53 °C, whose lamellae in AFM characterization display a grating architecture.

**Figure 9:**
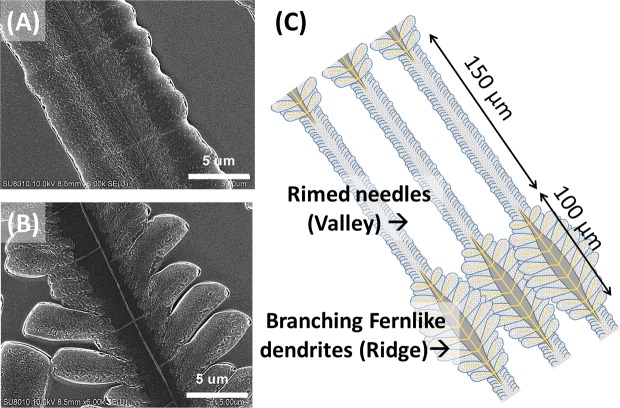
SEM micrographs for (**A**) straight rimed needles as “valley”, (**B**) fern-like branches as “ridge”, and (**C**) schemes for periodic-fractal banded pattern of PA/TA (80/20) dissolved in ethanol/water (20/80) crystallized at *T*_*c*_ = 53 °C. Band spacing = crossbar pitch = 150 + 100 = 250 μm.

Such grating-array bands are dramatically different from the conventional circular bands commonly seen in many other polymers or small-molecule compounds. Figure 9 shows SEM micrographs for periodic bands composed of two distinctively different morphologies: (A) rimed stalks (as ridge) and (B) fernlike-dendrite region (as valley), respectively, in PA/TA (80/20) crystallized at *T*_*c*_ = 53 °C. With more careful examination, one can observe that both the sector-like planes and the needle-like branches are composed of nano-size crystals, yet they are assembled in different way. For the sector-like planes in the fernlike dendrites region, there is adequate space to grow due to the loose branching in the initial stage. Therefore, the nano-size crystals can disperse averagely and arrange along the growth direction of dendrites. By contrast, for tiny needle-like branches, there is insufficient space to grow owing to the intensive branching in the initial stage. Thus, the nano-size crystals have no space to develop and can only arrange dominantly along the growth direction of the main stalks. In addition, the plentiful space allows sector-like plates to expand and branch out in 75°-angle direction from the main stalk. Owing to the lack of available space, compact needle-like branches overlap with each other and branch out in the perpendicular direction from the stalk. Schematic cartoon in Fig. 9C shows that the alternate optical bands correspond to lamellae of slender rimmed stalks of 170 μm in length and fern-like branched dendrites of 110 μm in length, respectively. Upon close inspection, the rimmed stalks (Fig. 9A) actually are packed with numerous wide-leaf branches of very short length; thus this region appears to be “rimmed” with zig-zag saw teeth. These short branches grow outward from the main stalks at ~90° angle. Oppositely, the fern-like branches (Fig. 9B), intersecting with the main stalk at 75° angle, display no twist and remain flat-on. These two regions alternate in periodicity, with an assembled “crossbar pitch” equal to 150 + 100 = 250 μm. The optical banding apparently is not due to lamellae continuously helix-twist, but is a result of grating architecture composed of two alternate morphologies of slender rimmed stalks and wider fern-like branches.

**Figure 10:**
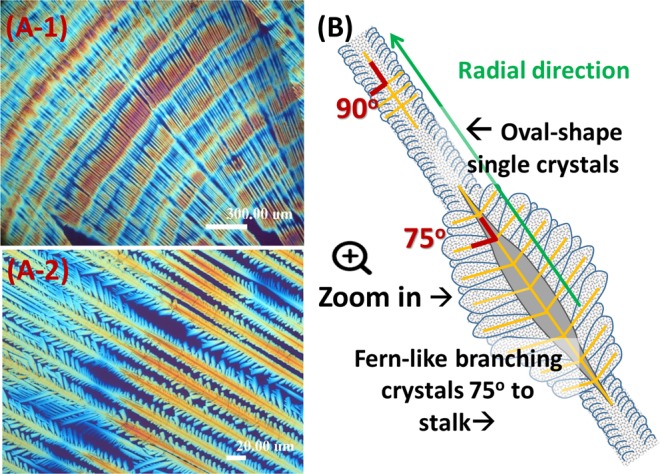
(A-1,2) POM graphs for grating-bands in PA, and (**B**) scheme of an individual lamella of two alternate morphologies along same stalk, representing the main characteristic of banding pattern with a non-twist grating packed by fractal-shape lamellar assembly in PA/TA (80/20) blend crystallized at *T*_*c*_ = 53 °C.

To sum up the main features of periodic fractal-branch banded pattern in terms of the correlation of the crystal assembly with the alternating optical birefringence bands, a scheme for PA/TA (80/20) crystallized at 53 °C was made to simplify the description. Figure 10 shows that the banded pattern consists of numerous repeated fractals periodically packed into a grating structure. Every fractal cycle is composed of a stalk and fernlike dendrites, displaying contrasted birefringence. The main stalk contains tiny needle-like branches growing densely in the perpendicular direction from the lateral sides due to crowded space. Thus, the nano-size crystals constituting the needle-like branches have no choice but to align along the original direction of the stalk and demonstrate optical positive birefringence (orange colour). By contrast, the fernlike dendrite comprises sector-like planes branching out loosely at almost 75° angle orientation from the stalk owing to better sufficiency of available space. Therefore, the nano-size crystals constituting the sector-like planes can align perpendicularly to the growth direction of the main stalk and thus such a grating structure with crystals aggregating and intersecting at 75° angle displays an optical contrasted negative birefringence (blue colour) differing from that of the main crystal stalk. It clearly suggests that the periodic grating structure without any crystal-plate twisting is packed into repeated growth fractals, which are the main characteristics leading to the formation of this unique periodic banded pattern.

Note that the fractal patterns of the solution-cast small-molecule PA are very similar to the fractal nanostructures in silk sericin (a long-chain protein made of 18 different amino acids) cast on mica as reported by Khire and Yadavalli, *et al*.^4^ These facts suggest that the self-similar and repetitive fractal patterns in crystal aggregates tend to be universal and are independent of chemical structures being either small molecules or long-chain polymers, synthetic compounds or natural macromolecules. Furthermore, similar branching packed in the hierarchical structures of banded polyethylene (PE) spherulites was reported by Toda, *et al*.^34^, who modelled the lateral width (λ) and length (gλ) of the branches, and the oriented slant angle (Δθ) between the stacked branches in banded PE. Their discussion was based on the branching instability of growth front and torsional stresses by following Bassett, *et al*.’s treatments of crystallization reorganization of folding surface and thickening^35,36^. However, in the case of grating banded PA spherulites with fractal branches, it is hard to come up with rationale that the lateral width and length of the branches along the main stalks vary periodically. Perhaps, the branching instability of growth front of the PA/TA (80/20) mixture periodically fluctuates in cycles dependent on diffusion replenish and drainage.

### Grating patterns at high temperature

**Figure 11:**
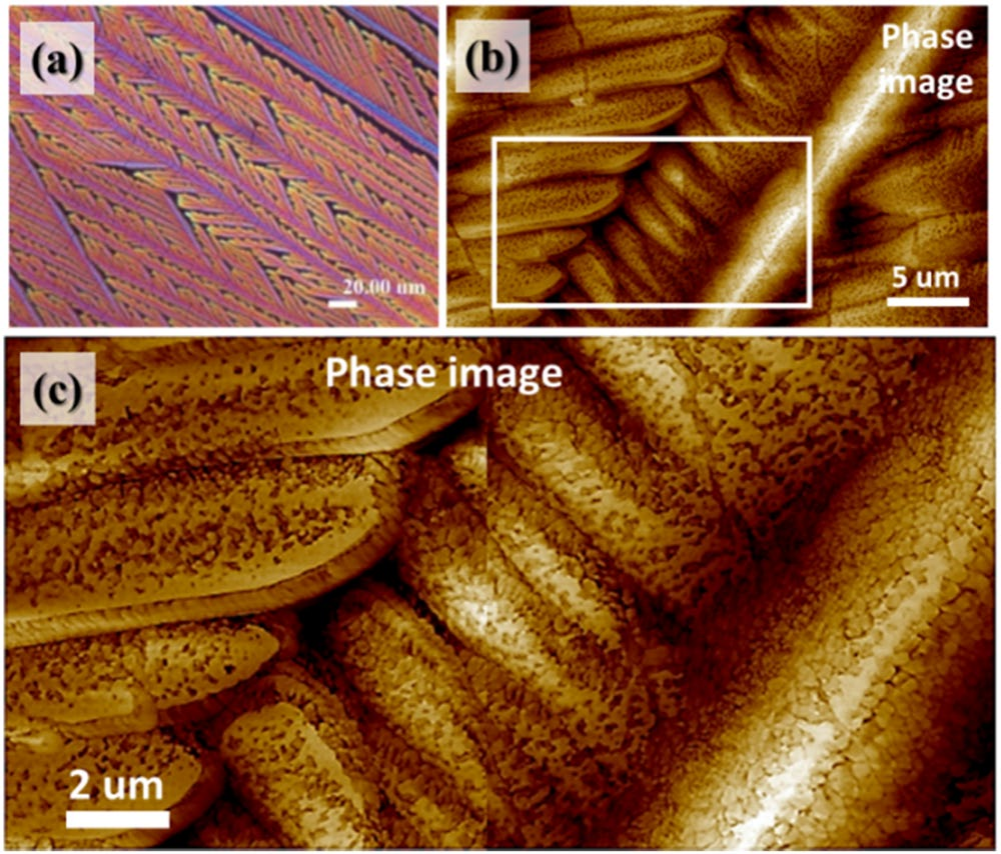
(**a**) POM image, and (**b**) AFM phase image, (**c**) zoom-in to square in Graph-(b) of grating pattern of PA/TA (80/20) dissolved in ethanol/water (20/80) crystallized at 60 °C.

An even higher *T*_*c*_ = 60 °C was used to produce PA crystals by crystallization from the same PA/TA mixture (80/20) dissolved in the same dual co-solvents. The periodic bands are still based on grating structures with periodic branches; however, the branching angle is no longer 75–90° as that crystallized at *T*_*c*_ = 53 °C. The branching angle is lowered to 60° instead. Figure 11 shows POM and AFM images of grating patterns of PA/TA (80/20) crystallized at *T*_*c*_ = 60 °C. All of the main stalks grow continually forward along the same direction instead of forming a spherulite, which is similar to the fractal-branch banded patterns crystallized at 53 °C. Nevertheless, different from the periodic fractal-branch banded patterns, grating patterns show highly ordered branches evolving consistently from their main branch, displaying the same optical patterns, as shown in Fig. 11(a). In addition, all main stalks and side branches mutually intersect with approximately 60° angle. The main stalks grow monotonously along a specific direction, parallel to each other, and the interval is 30–35 μm. The side branches further grow uniformly with branching angle 60° from the main stalk. Each side branch is ca. 2 μm in width and develops pointedly to fill the space between neighbouring stalks. Random finer and shorter branches grow from the side branches if the space allowed. The well-organized and linearly-aligned branches constitute the grating with hierarchical structures composed of main stalks and branches, which in turn are packed with aggregation of single crystals. AFM images show that both main stalks and side branches are composed of nano-size crystals, as shown in Fig. 11(b,c), which are similar to the structure of periodic branches of fractal-branch banded patterns crystallized at 53 °C.

**Figure 12:**
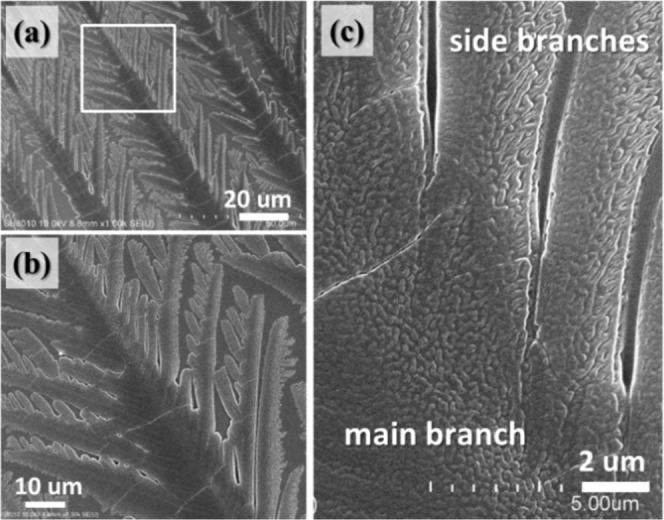
SEM micrographs (**a–c**) with increasing magnification as indicated on the graphs revealing the grating-structure lamellar pattern of PA/TA (80/20) in ethanol/water (20/80) crystallized at *T*_*c*_ = 60 °C.

In addition to POM and AFM analyses, Fig. 12 shows SEM micrographs for the detailed crystal assembly of branching structure with grating patterns. The primary side branches grow symmetrically from both sides of the main branches, and the secondary side branches with a finer structure evolving from the primary side branches if space allows. All the branches mutually intersect with main stalks at a constant angle of 60°, which may be governed by the geometry of the PA’s crystal lattices. The angle of interaction, though usually 60° angle, between the main stalks and branches, however, can be altered by factors such as crowdedness of the branches or stresses in crystal impingement during growth. Note that the branching pattern is highly symmetrical and the widths of the primary side branches are almost constant. The zoom-in image of the branches emerging region reveals all the branching pattern is packed by nano-size crystals, where they are parallel with each other along the growth direction of the main stalk. As the side branches develop, the nano-size crystals gradually tilt away from the original direction, thereby align themselves at 60° to the main stalk. Subsequently, the nano-size crystals grow forward and slightly branch out at an angle of 60° to fill the ever expanding space between the neighboring branches and further form the complete side branches. Note that when the crystal growth is not overly crowded/jammed, the side branches tend to be 60° angle consistently with the mother crystals from which the branches evolve. SEM graph in Fig. 12b (zoom in from Fig. 12a) shows clearly that parallel-aligned side branches (ca. 40 μm) are symmetrically on both sides of the main stalk (diagonal line) intersecting at 60° angle to each other. Then, from these side branches, there are tertiary “grand-daughter” branches (ca. 5 μm in length), intersecting again at 60° angle to the secondary (daughter) branches.

**Figure 13:**
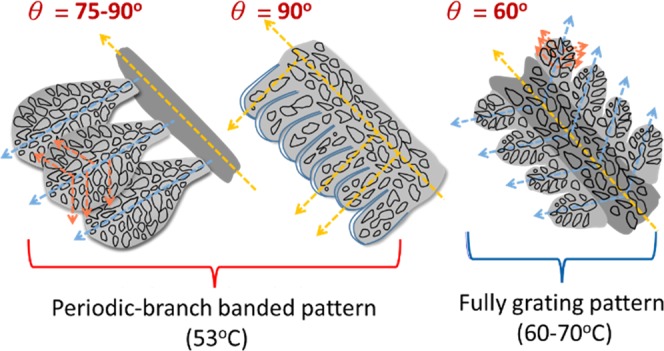
Branching angle variation with *T*_*c*_ (temp. of casting): (**A**) periodic-branch bands (*T*_*c*_ = 53 °C), (**B**) fully grating (*T*_*c*_ = 60–70 °C) in PA/TA (80/20) dissolved in ethanol/water (20/80) crystallized at 53–70 °C.

Schematic illustration is shown in Fig. 13 to simplify the main characteristics of crystal packing in the grating structure of periodic PA bands in PA/TA (80/20) blend crystallized at 53–70 °C, where the growth direction of the main stalk and side branches are specified, respectively. According to previous SEM results, the main stalks and branches are composed of all nano-size crystals growing in specific directions. With continuing development of crystals, well-organized side branches, with almost same size, branch out simultaneously at an angle of 60° from the main stalk. The orientation of the corresponding crystals changes into an angle of 60° to the growth direction of the main stalk. The identical and highly symmetric branching patterns leads to the formation of the ordered grating structure. The angle of branches (60° or 90°) with mother stalks is approximately in agreement with the crystallographic angles of PA. The crowdedness in branches tend to determine what dominant intersection angles will be. In loosely packed crystal at *T*_*c*_ = 60–70 °C, the angle tends to be ~60°, while in highly crowded branches (*T*_*c*_ = 53 °C), the angle of intersection can be 75 ° in the fern-like branches or ~90° angle in the stalk-rimmed branches. Note that the branching angle of 60° is with respect to a hexagon shape in crystal facets and lattices. However, due to the crowdedness or impingement in neighboring lamellae (single crystals) during growth, the branching angle may be displaced to any angles between 60° to nearly 90°.

When confined in micrometre-thin films, many small-molecule organic or inorganic compounds are known to form ring bands upon melt- or solution-crystallization^7–9,25–30,37^, as well as many long-chain polymers^18,38–40^, although the mechanisms driving the formation of such periodic rings have been controversially debated for many decades till now. Even hybrid inorganic compounds with polymers can form periodic bands. Kato *et al*.^41^ recently synthesized organic-inorganic hybrid compounds of hydroxyapatite (HAP)/poly(2-hydroxyethyl methacrylate) spin-cast as thin films on treated glass slides, and they proved formation of periodic rings with band spacing of ~3 μm. For long-chain polymers with chain folding back and forth in the lamellae, many investigators intuitively attributed the formation of periodic rings in polymers to lamellae being helix-twisted into a continuous screw-like thread by the “surface stresses” due to chain folding^42–44^. However, ample studies in the literature as cited have pointed out abundant cases of formation of same or similar ring bands in crystallization of many small-molecule compounds^7–9,25–30,37^ that lack chain-folding, thus no surface stresses for maintaining continuous helix twist, in their lamellae. Therefore, it must be cautioned that attribution of ring bands to chain-folding induced stresses may be over-simplified or it may be just coincidental occurrence. In reality, formation of such periodic rings may be driven by many complex factors that differ from one system to another, although they may share some common traits of crystal habits.

## Conclusions

The crystal assembly of banded morphologies, including ring-banded spherulites and fractal-branch banded patterns, reveal similar periodic branching features leading to the formation of optical bands, which demonstrates that the formation of periodic branches is the key characteristic of banded patterns. In addition, all of the branches in fractal-patterns with a grating structure are packed by nanosize crystals, albeit assembled in different ways or orientations depending on the density of branching in available space. These facts clearly suggest that the accountable growth mechanism of the novel fractal-branch banded PA crystals combines the main features of banded spherulites and grating patterns, and there exists some delicate correlations between the periodic bands and crossbar grating (dendritic) structures. That is to say, continuous lamellae of monotonously screw-like helicoids radiating outward without any branches from a nucleus centre are not likely and never fully supported by direct morphology evidence. Furthermore, undisrupted continuity in lamellae helix-twist with screw pitch matching optical band spacing has never been proved in the literature in past many decades. Instead, periodic branching with a grating structure is common as a driving mechanism in formation of periodic bands and periodic branching in fractal repetition is essential to fill the growing space, and the crossbar pitch of the mesh-like grating structure perfectly matches with the optical inter-band spacing.

## Methods

### Materials and preparation

**Scheme 1:**
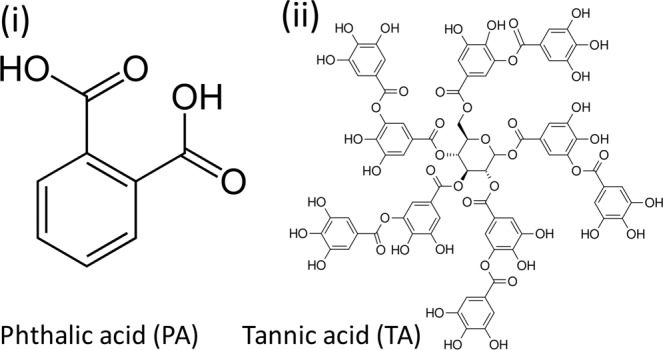
Chemical structures of (i) PA and (ii) TA. PA structure re-drawn from Own work, Public Domain, https://commons.wikimedia.org/w/index.php?curid=1337367 ^45^. TA structure – re-drawn from en:User_talk: Ronhjones - Own work, Public Domain, https://commons.wikimedia.org/w/index.php?curid=7685367 ^46^ (*The copyright holder of this work releases this work into the public domain and grant anyone the right to use this work for any purpose*).

Phthalic acid (PA), a small-molecule organic compound with di-acids on meta-position of benzene ring, was purchased from Alfa Aesar, Inc. (USA), with molecular weight (MW) = 166.13 g mol^−1^, *T*_*m*_ = 205 °C. Tannic acid (TA), a multi-phenol compound, was purchased from Aldrich Chem. Co. (USA), with MW = 1,720 g mol^−1^. The chemical structures of PA and TA are shown in Scheme 1. Binary mixtures of PA and TA of different compositions (80/20 to 20/80 wt. ratios) were first dissolved in the mixture of ethanol/water solution (of various vol. ratios) with concentration of 0.12 M, using the method in accordance with previous investigations on neat PA^29,31^. Film samples were prepared by direct casting one drop of PA-TA solution on a glass slide and placed on a hot stage pre-set at a specific temperature until the solvent was completely evaporated. The amount of droplet (cast on glass slides) was one drop, which was ca. 0.05 mL. The film thickness usually ranged from 3–5 μm (measured using film thickness analyzer). During the solvent evaporation from the PA/TA solution, PA rapidly crystallized while TA remained as an amorphous diluent. Specimens (solutions of PA/TA in co-solvent of various volume ratios), as one droplet, were cast on glass slide placed on the microscopic hot stage at isothermal *T*_*c*_’s (40–100 °C) till they were fully crystallized. Note here that solvent evaporation was complete within ca. 1 min, and once evaporated, the crystal growth of PA virtually was completed in the last stage of solvent evaporation. The final morphologies of all systems at T_c_ = 40 °C (or any other higher T_c_’s) in co-solvents were set within a few seconds. That is, upon solvent evaporation, the crystal growth of PA was just extremely too fast to be feasibly quantified.

Objective of incorporating TA during PA crystallization was specifically designed. In order to modulate different morphologies of PA crystals, PA was diluted with strongly-interacting TA. In addition, evaporation temperature and the compositions of mixture of ethanol/water solvents (ethanol/water = 20/80 to 80/20) were also two influential factors for modulation of PA morphology upon solution evaporation. These factors were manipulated to control the crystallization kinetics to modulate the PA morphologies for further analysis.

### Apparatus and procedures

A polarized-light optical microscopy (POM, Nikon Optiphot-2), equipped with a Nikon Digital Sight (DS-U1) camera control system and a microscopic hot stage (Linkam THMS-600 with T95 temperature programmer), was used for crystallization and characterizing the crystalline morphology. Quantification of fractal patterns of crystallized PA in POM micrographs was performed. By using image analysis, colour POM images of the grating structures were first converted to binary (B&W) images and analysed with a box-counting method as proposed by Lin, *et al*.^32^ The estimation procedures of the box-counting method for measuring the fractal patterns of crystallized PA are placed in the supporting information (ESI).

High-resolution field-emission scanning electron microscopy (HR-FESEM, Hitachi SU8010) was used to characterize the detailed assembly lamellae or lamellar bundles in crystallized PA spherulites from PA/TA mixtures. Solution-cast samples were coated with platinum using vacuum sputtering (10 mA, 300 seconds) prior to SEM observation.

Atomic-force microscopy (AFM) investigations were made in intermittent tapping mode of AFM (diCaliber, Veeco Co., Santa Barbara, USA) with a silicon-tip (f = 70 kHz, r = 10 nm). AFM measurements were mainly used to investigate the top surfaces for probing the crystalline lamellar arrangement on surface-relief topology as well as analyses of the height profiles across the surfaces.

## Supplementary information

Supplementary Information.
