# Supplementary material for: Periodic Fractal-Growth Branching to Nano-Structured Grating Aggregation in Phthalic Acid
Source: Sci Rep. 2020 Mar 4;10:4062. doi: 10.1038/s41598-020-60782-0 (PMC7055272; doi:10.1038/s41598-020-60782-0)
Supplement: Supplementary file 1 — Supplementary Information. [file 41598_2020_60782_MOESM1_ESM.docx]

Periodic Fractal-Growth Branching to Nano-Structured Grating Aggregation in Phthalic Acid

Tzu-Yu Chen, Eamor M. Woo*, and Selvaraj Nagarajan

Department of Chemical Engineering, National Cheng Kung University

No. 1, University Road, Tainan, 701-01, Taiwan

**Supporting Information**

**Figure S1.** POM micrographs for PA/TA mixture of various compositions: (a) 100/0, (b) 90/10, (c) 80/20, and (d) 70/30, all crystallized at same *T_c_* = 70°C.


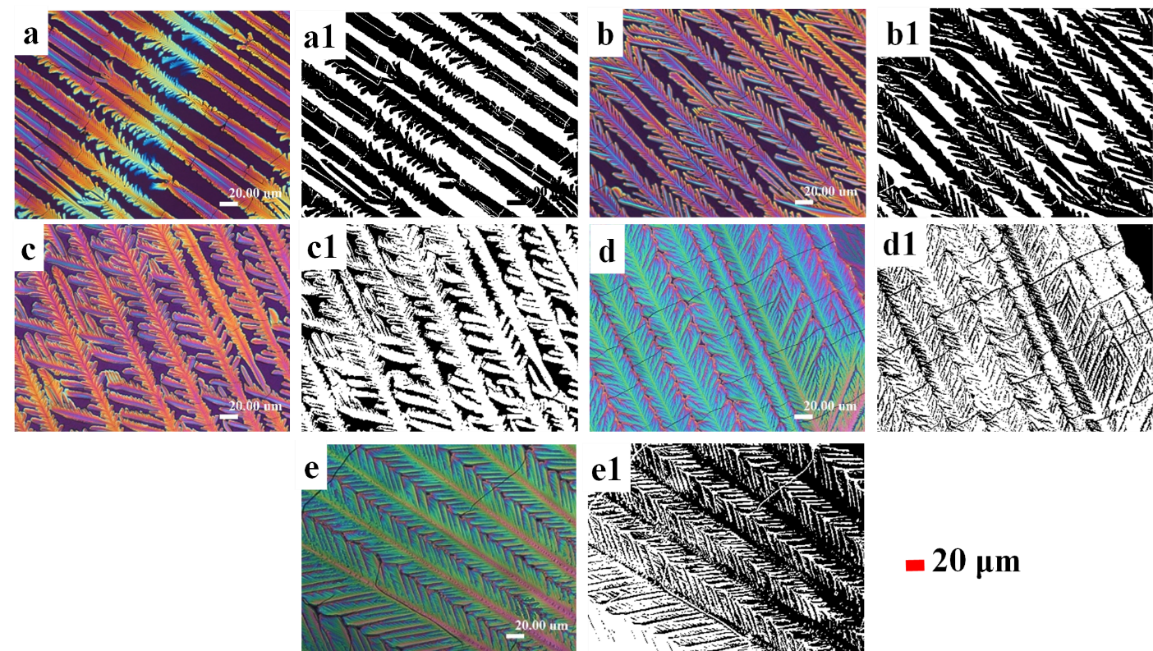


**Figure S2:** Box-counting method procedure for fractal dimension D of POM results of PA/TA (80/20) blend dissolved in different compositions of ethanol/water solution. (a) 45/55, (b) 50/50, (c) 60/40, (d) 70/30, and (e) 80/20 corresponding binary images (a1-e1).

**Fractal Dimension of Grating Structures**

Using POM grating structure image of PA/TA (80/20) blend dissolved in different compositions of ethanol/water given in Figure S3(a-e). The binary image that correspond to the POM shown in Figure S3(a1-e1). Using binary images, we estimated the fractal dimension of the grating structure.

For image analysis box count method used to compute the grating structures. Fractal dimension (D) is calculated using equation 1 and 2.**^36,37^**

$\underset{\lambda\to0}{D=-lim} \frac{ln(N(\lambda))}{ln(\lambda)}$ (1)

Where: **(λ) box length, N(λ)- number of boxes** required to cover the grating structures, and it expressed as:

Ln(N (λ)) = -D ln(λ) + constant (2)


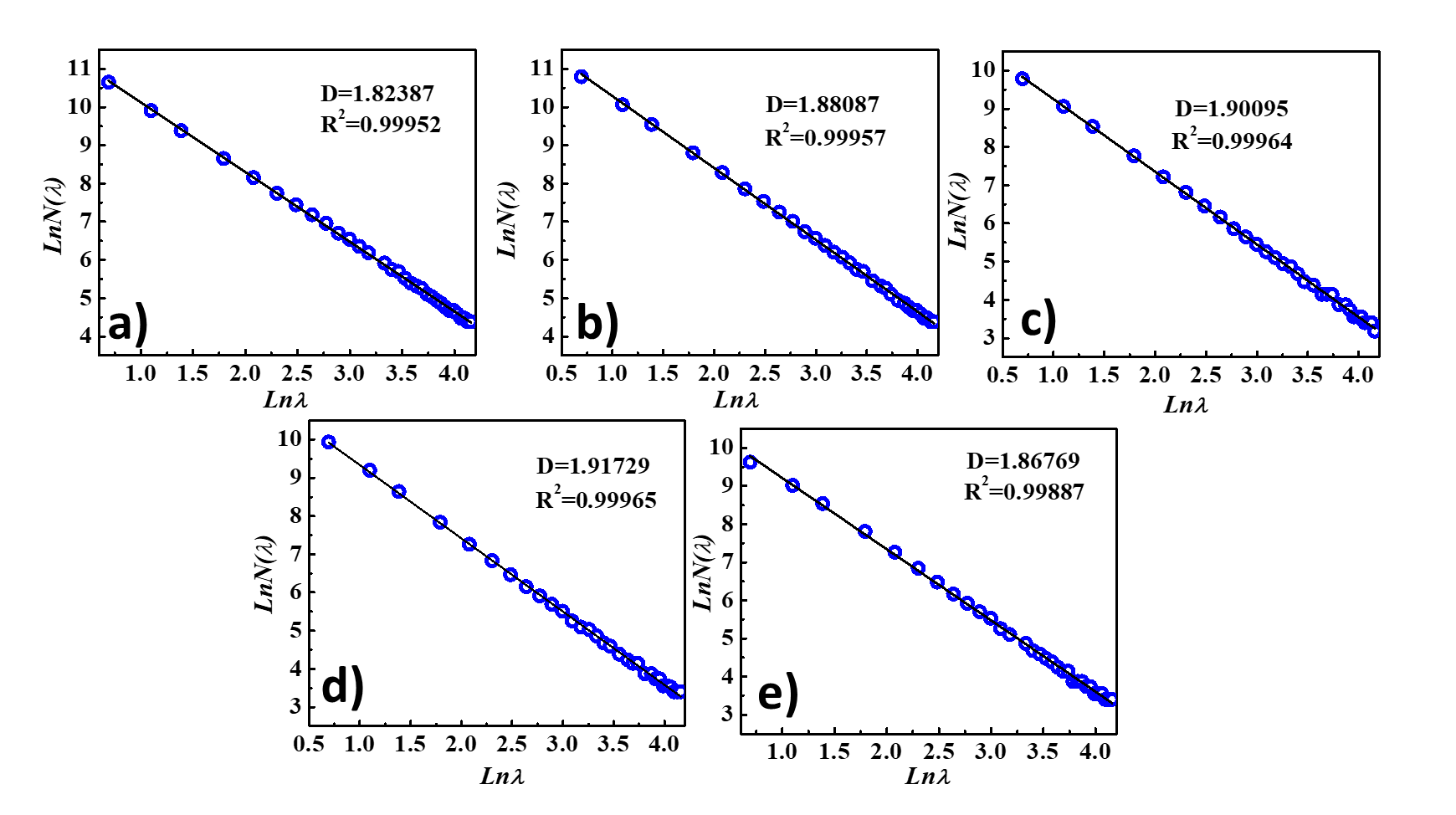


**Figure S3:** Fractal dimension calculation results from POM image analysis: blue circles indicate the data point and block line indicate linear line fit. PA/TA (80/20) blend dissolved in different compositions of ethanol/water solution: (a) 45/55, (b) 50/50, (c) 60/40, (d) 70/30, and (e) 80/20.


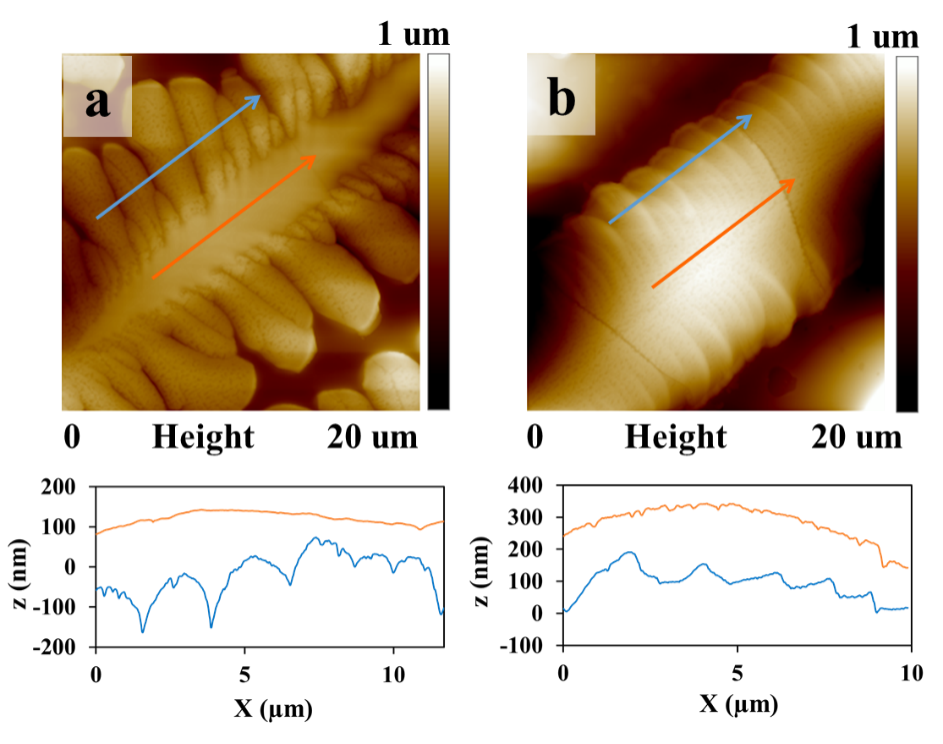


**Figure S4.** AFM height images and height profiles of (a) fernlike-branch dendritic region and (b) stalk rimmed with zig-zag short branches of grating banded PA/TA (80/20) dissolved in co-solvent ethanol/water (20/80) crystallized at 53°C.

References (same # as in main texts)

36. Lin, K., Huang, X. & Zhao, Y. Combining Image Recognition and Simulation To Reproduce the Adsorption/Desorption Behaviors of Shale Gas. *Energy & Fuels* **34**, 258–269 (2020).

37. Hu, Y. Q., Zhao, Y. P. & Yu, T. X. Fractal pattern formation in anodic bonding of pyrex glass/Al/Si. *Int. J. Nonlinear Sci. Numer. Simul.* **9**, 315–322 (2008).
